# Low density phases of TiO_2_ by cluster self-assembly

**DOI:** 10.1038/s41598-024-61943-1

**Published:** 2024-05-31

**Authors:** Faustino Aguilera-Granja, Andres Ayuela

**Affiliations:** 1 Instituto de Física, Universidad Autónoma de San Luis Potosí, 78000 San Luis Potosí, Mexico; 2 Centro de Física de Materiales-CFM-MPC, Donostia International Physics Center DIPC, Paseo Manuel de Lardizabal 5, 20018 San Sebastián, Spain

**Keywords:** Materials science, Nanoscience and technology, Physics

## Abstract

The interest in titanium dioxide (TiO_2_) phases is growing due to the number of applications in cosmetics, food industry and photocatalysis, an increase that is driven by its exceptional properties when engineered at the nanoscale like in the form of nanoparticles. Our goal is to discover unknown low-density phases of TiO_2_, with potential for applications in various fields. We then use well-known TiO_2_ clusters as fundamental building blocks to be self-assembled into unique structures to study their distinct characteristics. Density functional calculations are employed to relax the structures and identify the most stable TiO_2_ structures within an energy range of 0.1 eV per atom from the rutile and anatase phases, which are confirmed, validating our methodology. Going beyond conventional phases, we found two-dimensional TiO_2_ structures, previously explored in separate studies, and showing typical structures of transition metal dichalcogenide layers, that forge a bridge between different TiO_2_ structures. It is noteworthy that our investigation uncovered an entirely novel class of TiO_2_ structures featuring hexagonal cages like beehive channels, opening novel phases with huge potential. These discovered low-density phases are interesting, particularly the hexagonal cage structures with remarkable large gaps, because they introduce other dimensions for uncharted applications in the ever-growing TiO_2_ landscape.

## Introduction

**Figure 1:**
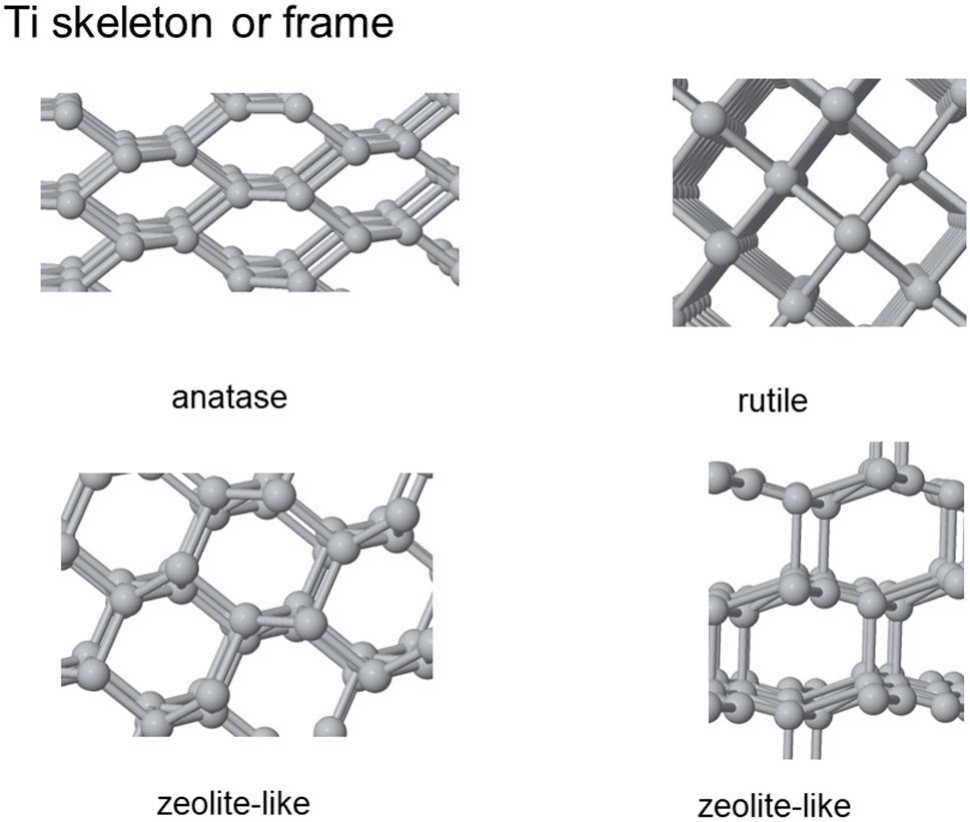
Ti skeleton within some selected phases generated using small cluster seeds.

Within materials science, titanium dioxide (TiO_2_) has long receive the attention of researchers due to its diverse polymorphs with exceptional properties^1^. Key applications of TiO_2_^2,3^ are found in industries such as in cosmetics as UV protection, in food industry as food additive and whitening agent, and in photocatalysis for environmental remediation similarly to other materials^4,5^. Bulk TiO_2_ phases based on the conversion of light into chemical processes have extensive applications spanning photocatalysis^6,7^, solar photovoltaics^8,9^, biomedicine^10^, and environmental remediation^11,12^ and they remain currently an ever-evolving field of study. However, in the quest for novel TiO_2_ phases, an intriguing avenue of exploration has emerged under high-pressure conditions with high densities^13–18^. While TiO_2_ nanostructures being more compact have been extensively studied in various contexts^19^, like nanoparticles, small clusters of TiO_2_ synthesized under low density conditions presents a unique and unexplored realm^20^. Self-assembly of clusters presents today an innovative approach^21^, with a distinct behavior observed at low densities, and offers the potential to uncover new TiO_2_ phases, as schematically shown by the skeleton of Ti ions in Fig. 1, enriching the fundamental understanding of this remarkable material while holding great promise for applications.

As we delve into the intricate world of TiO_2_ phases, it becomes evident that rutile, anatase, and brookite are the most prominent crystalline forms, each endowed with distinct structural characteristics and properties^1,22^. Rutile, well-known for its remarkable stability^23^, finds applications in optical coatings^24,25^ and high-performance ceramics^26,27^. Anatase, with its enhanced photocatalytic properties, is extensively employed in environmental remediation and solar cell technologies^28,29^. Brookite, although less common, exhibits unique electronic properties, making it a subject of increasing interest for novel electronic devices^30,31^. All the phases have titanium ions octahedrally coordinated to oxygen ions. The octahedra are arranged edge to edge in the anatase phase, and they show also corner to corner links in rutile and brookite phases, in the latter phase being piled in layers. Yet, the focus on rutile, anatase, and brookite has predominantly revolved around the high-pressure synthesis at higher densities^1,32,33^, research gap exists regarding the behavior of these phases at both higher and lower densities. Investigating these extremes in density can lead to a more comprehensive understanding of TiO_2_ phases and open doors to the tailored design of phases that span a wider range of applications, ultimately bridging the existing knowledge gap in TiO_2_ materials.

The investigation of high-pressure phases in titanium dioxide (TiO_2_) has ushered in a transformative era within materials science^34^. Under extreme pressure conditions, TiO_2_ exhibits a diverse array of polymorphic transformations, and results in the discovery of novel phases that challenge our conventional understanding of this versatile material^1^. At elevated pressures, rutile, anatase, and brookite, the three primary phases of TiO_2_, undergo remarkable structural transitions^13,35,36^. Previously stable rutile, renowned for its robustness, transforms into entirely unexpected phases such as baddeleyite, TiO_2_OI phase and others with unique properties as density increases^37–39^. This exploration into high-pressure and high-density phases has not only broadened our comprehension of TiO_2_ but has also paved the way for potential applications in cutting-edge technologies, from high-pressure environments to advanced energy storage systems^40–43^. Note that in this research, there remains an intriguing gap in our understanding of low-density phases of TiO_2_, an area that promises to further enrich our knowledge of this remarkable material.

TiO_2_ small clusters have emerged as an interesting frontier in materials science research. Other stoichiometries based on the Ti/O ratios can be experimentally found^44^; however in rich O2 atmosphere the stoichoimetric clusters TiO_2_ are found to be much stable^45^, a fact that has been recently addressed from a theoretical point of view^46^. These small nanostructures exhibit unique properties and behaviors that diverge significantly from bulk TiO_2_. For instance, experimentally looking at the gap evolution with size, small clusters seems already close to those of rutile and anatase for (TiO_2_)_n_ with *n* larger than six^47^. Extensive theoretical studies have revealed that the properties of TiO_2_ clusters are highly dependent on their size and morphology^48–55^. In fact, exploring further the world of TiO_2_ clusters is fascinating from adsorption studies to the impact of metallic impurities on optical absorption properties^56–58^. Their remarkably high surface-to-volume ratio makes them promising candidates for various applications, from catalysis and sensing to photovoltaics^21,59^. Researchers have delved into the synthesis and characterization of these clusters, often exploring their behavior under extreme conditions, such as high oxygen pressures to probe low-density phases^45,47^. However, a new opportunity arises with the potential of depositing TiO_2_ clusters with specific sizes on surfaces^60,61^ and later self-assembling them into new phases^21,60,61^, particularly focusing on low-density environments. Optimization of reactive gas conditions is essential for tailoring the characteristics of transition metal clusters and their applications in nanomaterial synthesis for thin film deposition^21,60,61^. This study holds the promise of engineering novel phases, transforming the landscape of TiO_2_ materials.

In this work we employ density functional calculations to explore the self-assembly of TiO_2_ clusters and their potential to yield bulk structures of lower density. We aspire to contribute to the evolving landscape of TiO_2_ research and offer fresh insights into bridging the divide between high and low densities, ultimately advancing our knowledge of this exceptional material. Our research is not only a reflection of the profound versatility of TiO_2_ but also a pursuit to unlocking novel phases and horizonts in materials science. Then, we hope to inspire future investigations and open doors to expand the field within TiO_2_ research, broadening the understanding of this remarkable material and its potential applications.

## Results

**Figure 2:**
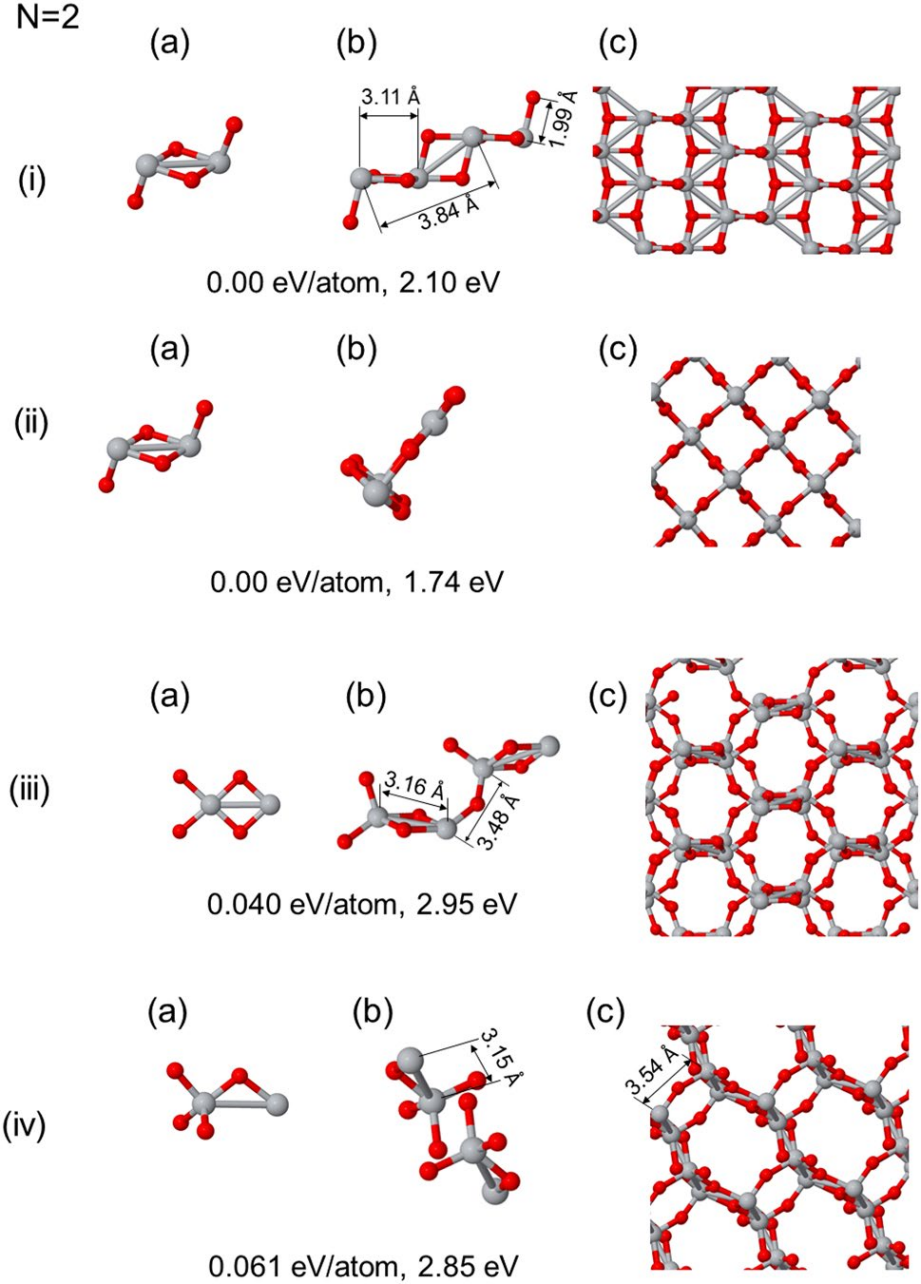
Different generated bulk-like structures for different (TiO_2_)_2_ pairs as seed clusters. In (**a**) the used seed is presented, in (**b**) the optimized atomic based cell, and in (**c**) a view of the generated bulk phase. Below each structure the energy difference with respect to the putative ground state and the HOMO-LUMO gap are included.

**Figure 3:**
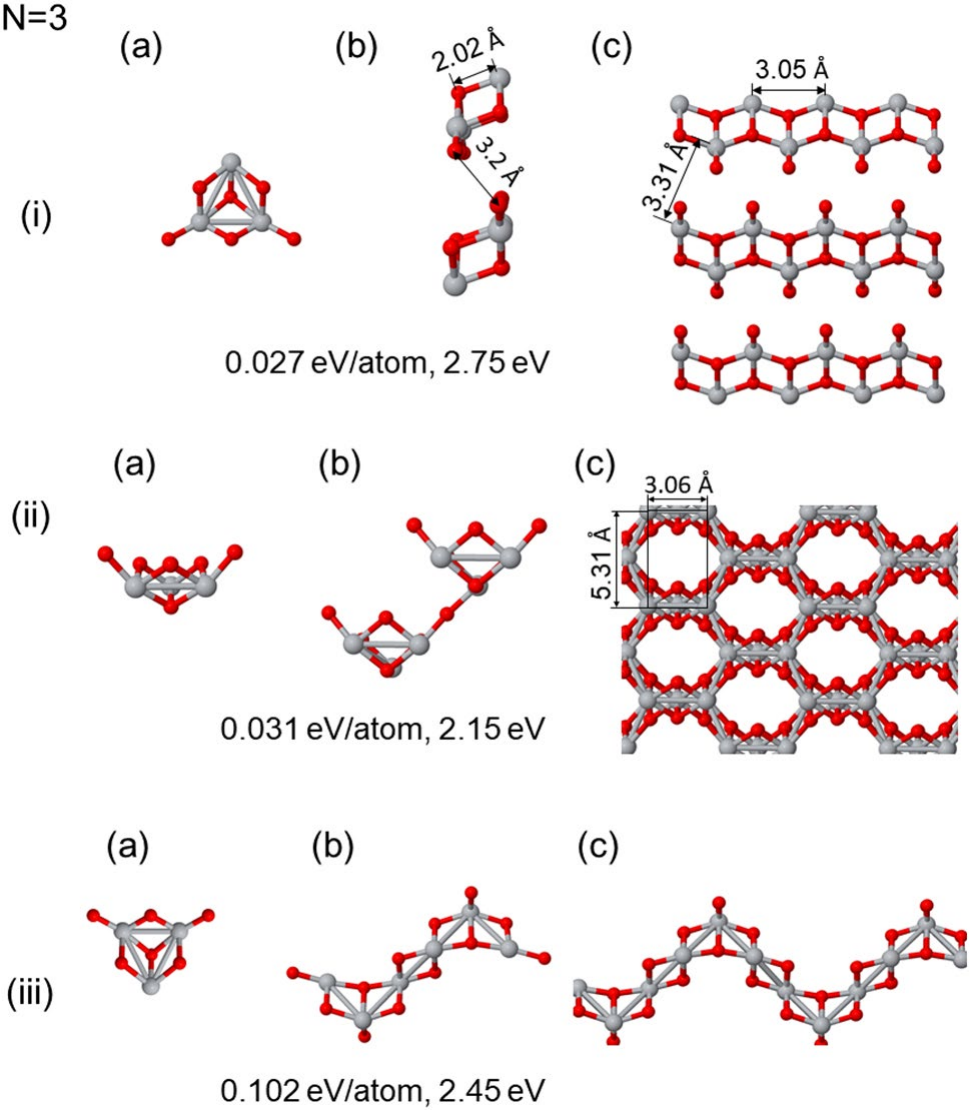
Generated bulk structures for the same seed (TiO_2_)_3_ (being the ground state cluster) in different directions in (i) to (iii). (**a**) Present the seeds used, (**b**) show the optimized atomic unit cells, and (**c**) provide a view of the corresponding generated bulk phase. The energy difference with respect to the putative ground state is shown below each structures, as well as the HOMO-LUMO gap.

**Figure 4:**
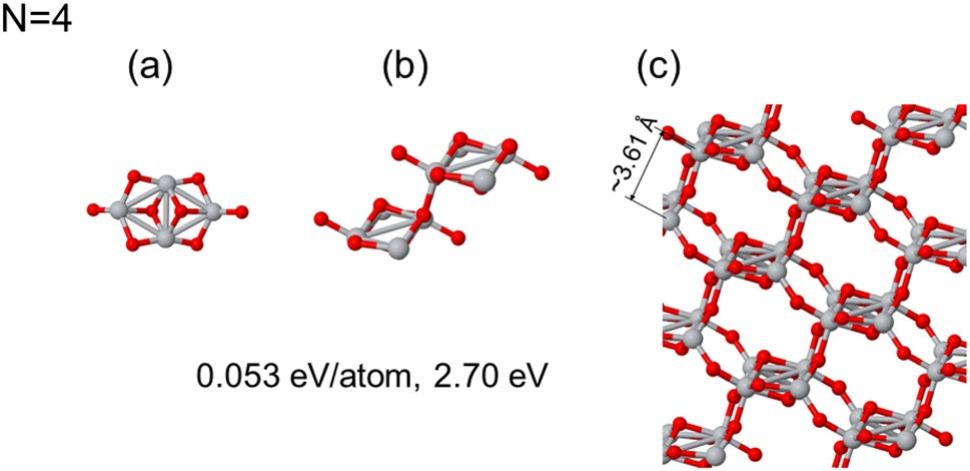
Bulk-like structure generated using (TiO_2_)_4_ ground state cluster as a seed. In (**a**) the used seed is presented, in (**b**) the optimized atomic based cell, and in the (**c**) panel a view of the bulk phase generated. The energy difference with respect the rutile bulk phase and the HOMO-LUMO gap are shown below.

**Figure 5:**
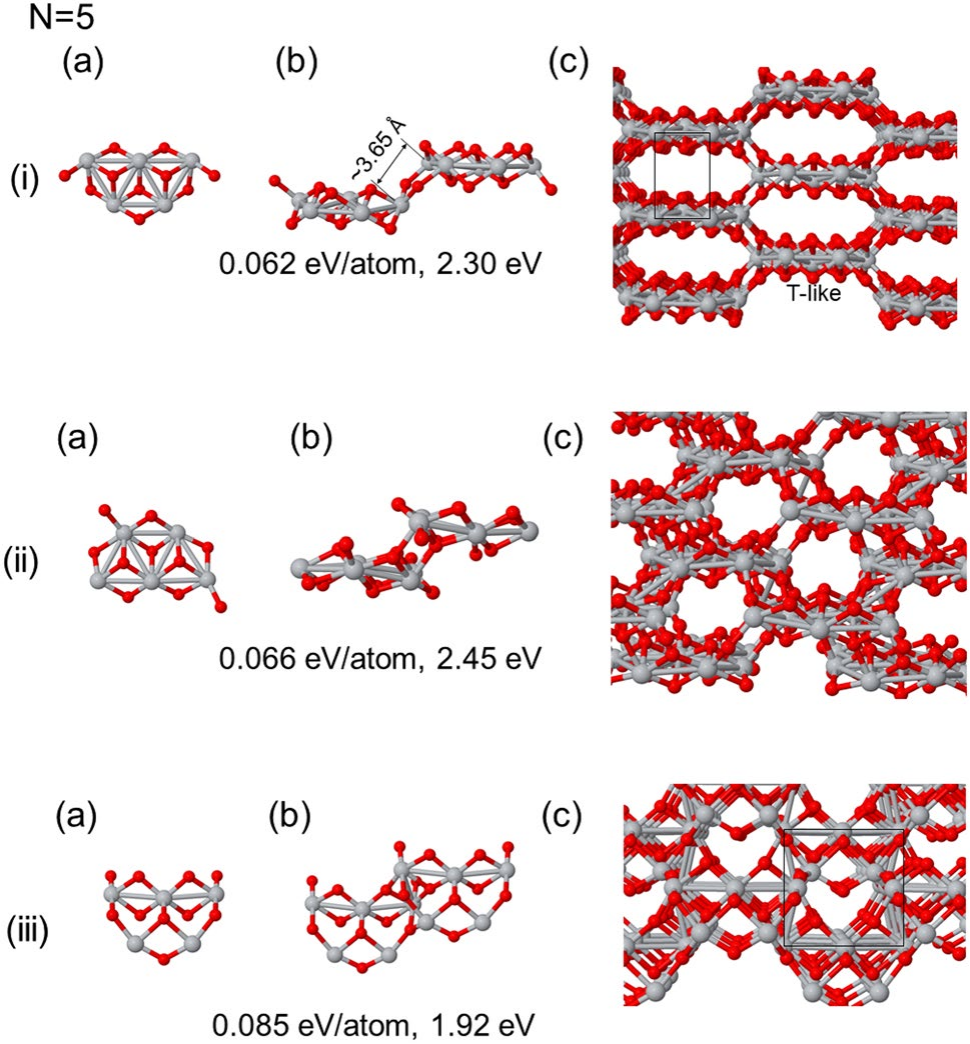
Bulk generated structures using different (TiO_2_)_5_ clusters. In (i) is the ground state cluster, (ii) and (iii) are nearby energy isomers. In (**a**) is presented the seed used, in (**b**) the optimized atomic based cell, and in the (**c**) panel a view of the bulk phase generated. The energy differences with respect the putative ground state bulk phase and the HOMO-LUMO gap are shown below each case.

**Figure 6:**
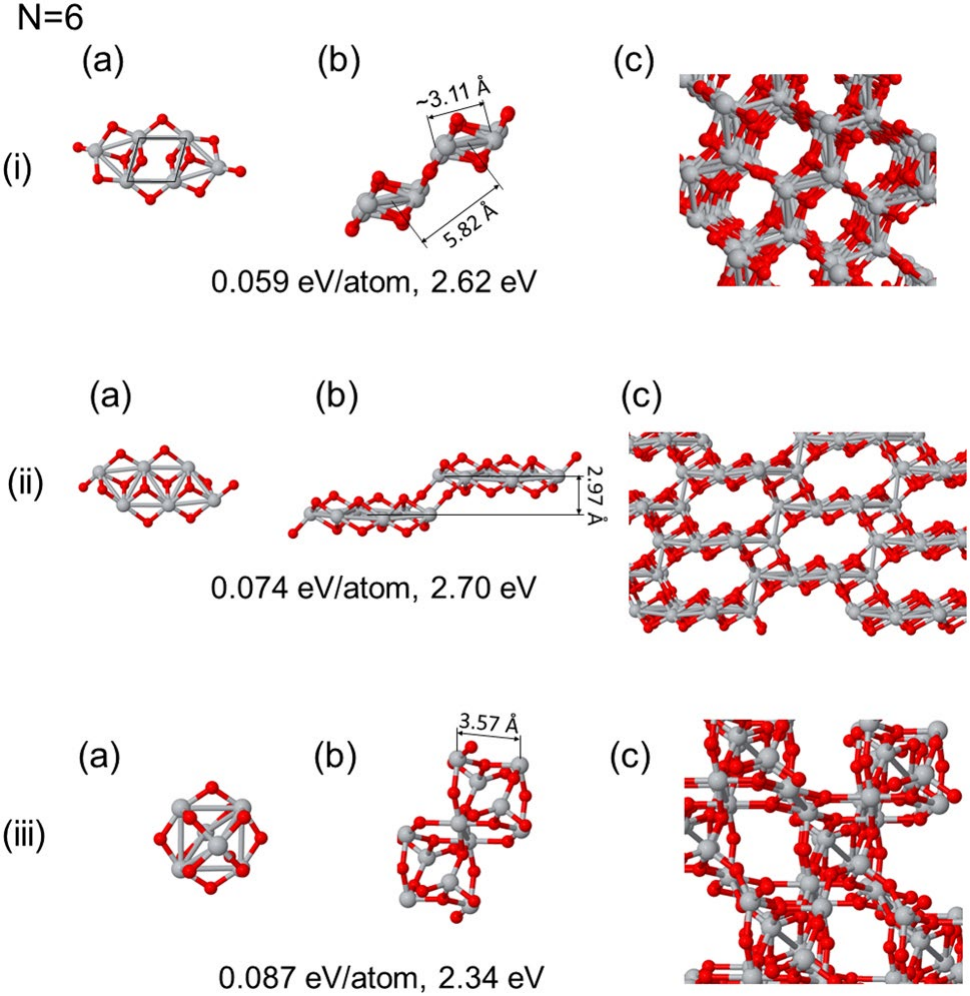
Bulk phase structures generated using different (TiO_2_)_6_ clusters as a seed, none of them are the ground state cluster. The seeds correspond to isomers close to the ground state. (**a**) Show the seed used; (**b**), the optimized atomic based cell; the (**c**) panels a view of the bulk phase generated. The energy differences with respect the putative ground phase and the HOMO-LUMO gap are shown in every case.

The core findings from our computational investigations are included through Figs. 2, 3, 4, 5, 6. It is noteworthy that the atomic cells used in our simulations are consequences of the initial seeds and the orthogonal character of the vectors (non-primitive vectors), possess a conventional-like structure. Within each of these figures, we present three distinctive states for each relaxed phase following the panels:

- corresponds to the seed employed in the calculation, serving as the foundational unit for the initial atomic base;
- represents the atomic base cell that emerges after optimization, highlighting the effects of self-assembly and structural refinement; and
- illustrates the bulk phase structure obtained by extending the optimized atomic base cell through periodic repetitions.

For a comprehensive understanding of these structures, including atomic coordinates, base vectors, their magnitude, volume, and atomic density, a detailed set of data is provided in the tables included in Supplementary Material. These figures and supplementary information collectively offer a rich insight into the outcomes of our calculations, and show the diverse structures and properties that arise from our studies. We here consider the structures that are 0.1 eV/atom from the most stable one.

### Bulk phases with (TiO_2_)_2_ cluster as a seed

The initial exploration of bulk phases using the (TiO_2_)_2_ cluster as a seed leds to the generation of two distinct structures. In the first case, as shown in Fig. 2(i), we obtained the anatase phase, characterized by specific interatomic distances. The Ti-Ti distances in this structure were measured at 3.11 Å for the first nearest neighbors, 3.84 Å for the first next nearest neighbors, and 4.93 Å for the third neighbors. The Ti-O distance was found to be 1.986 Å. These structural parameters align with a system having lattice vectors **a** and **b**, with values of 3.83 Å and 9.87 Å, respectively. These values are found to be less than 4% of what has been reported theoretically and experimentally, indicating a small deviation from existing data. In the second case, as shown in Fig. 2(ii), the generated structure corresponds to the rutile phase. The interatomic distances for this phase were measured at 3.01 Å for the first nearest neighbors, 3.61 Å for the first next nearest neighbors, and 4.65 Å for the third neighbors, with a Ti-O distance of 1.99 Å. These values correspond to the conventional lattice vectors **a** and **c**, which measure 4.67 Å and 3.02 Å for rutile, respectively, deviating by less than 2% from the theoretical and experimental values^62^. While both these rutile and anatase structures exhibit nearly degenerate total energies, the anatase phase appears to be slightly more stable in agreement with previous GGA calculations^55,63^. Furthermore the calculated HOMO-LUMO gap in both structures is underestimated, attributed also to the use of the PBE functional. The PBE values significantly differ from experimental values of anatase and rutile bulk phases. Nevertheless the PBE functional reproduces the rutile layer thickness more accurately than that of anatase. This calls for further refinement in PBE to provide an improved description of the relative stability of anatase and rutile phases in bulk TiO_2_ systems as investigated and commented below in the discussion section.

**Alternative open structures based on the 1st and 2nd low-lying isomers**

Exploring beyond the ground state, we also considered structures derived from the first and second low-lying isomers of the (TiO_2_)_2_ cluster. As depicted in Fig. 2(iii) and (iv), the seeds for these structures consisted of next lying isomers in energy, as shown in panels (a) for both cases. In Fig. 2(iii), we observed a low-density structure featuring channels with hexagonal symmetry. This structure exhibited specific interatomic distances, including Ti-Ti distances of 3.16 Å for the first nearest neighbors, 3.48 Å for the first next nearest neighbors, and 3.72 Å for the third neighbors, with a Ti-O distance of 1.997 Å. On the other hand, Fig. 2(iv) presented a structure characterized by a series of alternating planes with a Ti-Ti ziz-zag skeleton in planes, with a Ti-Ti atom distance distribution including values of 3.15 Å, 3.21 Å, 3.44 Å, 3.51 Å, and 4.43 Å. The zig-zag formation and displacement between Ti-lines in TiO_2_ structures studied in the research are part of a Ti framework to be decorated with oxygens. This arrangement optimizes the bonding and coordination of titanium (Ti) atoms with oxygen (O) atoms, reducing strain and enhancing structural integrity. In fact, the Ti-Ti zig-zag formation and displacement of Ti-lines in TiO_2_ structures can contribute to the overall stability and electronic properties of the crystal lattice. The minimum distance between the planes was measured at 3.54 Å. Additionally, two distinct types of Ti-O distances were observed, with inter-plane and intra-plane values of 1.988 Å and 1.853 Å, respectively. Remarkably, the HOMO-LUMO gaps and densities for both of these structures were quite similar. These structures exhibited open, low-density configurations that are similar to zeolites, indicating the emergence of novel structural forms within TiO_2_ compounds. This opens the door to further exploration and investigation of these intriguing structures in TiO_2_ materials.

### Bulk phases with (TiO_2_)_3_ cluster as a seed

In this section, we explore the diverse geometric structures achieved through the self-assembly of (TiO_2_)_3_ clusters, as displayed in Fig. 3. Panels (i) to (iii) show three distinct bulk phases using the ground state (GS) cluster of (TiO_2_)_3_ as a seed, positioned in different orientations. The GS clusters of (TiO_2_)_3_ exhibit a certain degree of polarizability and form remarkably open structures.

In Fig. 3(i), we observe the structure with the lowest energy, which follows a layered pattern similar to anatase or rutile. Bilayers composed of two rectangular Ti-Ti planes are prevalent, featuring intra-plane Ti-Ti distances of (3.05, 3.78) Å and an inter-plane distance of Ti-Ti = 3.31 Å. This structure incorporates additional bilayers arranged transversely to the previous ones. More importantly, the first neighbors exhibit a Ti-O distance of 2.02 Å, and an O-O distance of 3.20 Å is observed between neighboring capping oxygen atoms in bilayers. The density of this structure is similar to that of the anatase phase, although slightly lower (0.081 atom/(Å)^3^). In fact, the layers interact between O and Ti ions, mirroring the TiO_2_ structures previously found to be more stable in separate studies^64^. These structural units in different layers exhibit opposing polarizabilities, as shown in panel (b), which effectively cancel out to form the layered bulk phase.

Figure 3(ii) presents another bulk structure, characterized by the formation of bands (strips) arranged in an alternate pattern. These strips form rectangular shapes, with the shortest intra-plane Ti-Ti distance in the Ti-centered rectangle measuring 3.06 Å, and the longer side displaying Ti-Ti = 5.31 Å. Stripes at different levels are separated by Ti-Ti distances of 3.07 Å (non-orthogonal distance). The Ti-O distance exhibits an average of 1.995 Å with minimal dispersion, and the structure features a distinctive honeycomb-like channels. Furthermore, the structure revealed in panel (ii) represents an open configuration, also as a beehive on a lateral cross-section, while the walls in the x-direction resemble a so called “T”-like structure, similar to what has been described for transition metal dichalcogenides and explored in studies of transition metal oxides^64^. In this case, the ground state clusters form new bonds between the units, effectively cancelling polarizability locally.

In Fig. 3(iii), we show a quasi-two-dimensional structure spontaneously generated through the methodology described earlier. This structure consists of parallel Ti lines, featuring Ti-Ti distances of 3.03 Å. These lines, arranged in a triangular array, form a pattern with a periodicity of every three Ti lines from top to bottom, each exhibiting an average Ti-Ti distance of 3.10 Å. Together, they form a thin film of (TiO_2_). The spaces separating the different thin film images (slabs) exceed 8 Å, having them as independent systems. The case presented in Fig. 3(iii) exhibits an open configuration that maintains two layers with characteristic kinks. This structure effectively combines various motifs discussed earlier, with kinks resembling anatase structure and flat segments resembling the “T”-like structures. Combining these motifs seems to introduce an innovative path to attain highly stable open structures.

Lastly, we compare with a single layer of rutile type, to look at the energy cost associated with forming kinks and the displacement between Ti-lines (resulting in Ti-triangles). A genuine two-dimensional bi-layer system was studied in our previous work^64^. This system, similar to the basic array but in a rectangular form, can be created using the (TiO_2_)_2_ ground state as a seed. By comparing the energy of both cases, Fig. 3(iii) (0.102 eV/atom) and the single layer with 0.044 eV/atom, we conclude that the kink formation and the displacement between Ti-lines has an approximate energy cost of 58 meV/atom. Furthermore, we can see that piling single rutile layers to built the structure shown in Fig. 3(i) contribute with an energy gain about 17 meV/atom.

### Bulk phases with (TiO_2_)_4_ cluster as a seed

We now explore the bulk structure generated using the (TiO_2_)_4_ ground state as a seed, shown in Fig. 4(i). This particular cluster configuration exhibits a rhombic geometry in the skeleton formed by the Ti ions. It is important to note that other orientations of the seed result in bulk phases that either significantly deviate in energy or converge to those obtained before using the (TiO_2_)_2_ seeds.

The bulk structure originating from the (TiO_2_)_4_ seed is characterized by bands of Ti atoms, which takes on triangular shapes. This structure features an almost continuous distribution of Ti-Ti distances, measuring (3.03, 3.11, 3.20, and 3.80) Å. These Ti flat stripes are interconnected to other bands via Ti–Ti bonds in the range of 3.49 to 3.73 Å. The interatomic distances between Ti and O atoms range from 1.880 to 2.110 Å, with an average of 1.995 Å. Although the structure possesses periodicity, it becomes evident that the atomic base cell exhibits structural disorder, as given by the wide distribution of distances found. This structure has numerous channels of varying shapes in different directions, adding a remarkable degree of complexity to its three-dimensional configuration. Additionally, it shows the largest HOMO-LUMO gap (2.70 eV) among the structures considered in this study. When considering the two-dimensional stacked structure found in Fig. 4(i), the unique characteristics of this zeolite-like bulk structure becomes evident.

This configuration shares similarities with the open beehive structure described in the previous case in Fig. 3(ii). However, it is noteworthy to highlight that the seed in this instance does not exhibit polarization. It serves as another illustrative example of layered structures featuring the “T”-like motifs. In this case, it assumes an exceptionally open structure, which corresponds to its significant energy gap. An intriguing point of consideration is the slight difference in the atomic arrangement compared to the previous case in Fig. 3(ii), where triangular Ti atoms were shown. In the current configuration, a rhombic arrangement is observed. Although these structures may appear subtly different, they warrant closer examination, particularly regarding the placement of oxygen atoms. It becomes apparent that in this case, elongated platelets are formed to deform the beehive structure in a distinct direction, which provides a fresh perspective on the diverse array of structures attainable through self-assembly processes.

### Bulk phases with (TiO_2_)_5_ cluster as a seed

We here investigate the bulk phases generated by utilizing the (TiO_2_)_5_ cluster, including both the ground state and nearby isomers, as illustrated in Fig. 5. These isomers of TiO_2_ clusters can be understood as Ti atoms forming a skeletal structure decorated with O atoms. Note that this visual analogy helps in studying the diverse phases that emerge when these clusters are stacked together.

#### Self-assembly of (TiO_2_)_5_ ground state

In Fig. 5(i), we show the self-assembly of the (TiO_2_)_5_ ground state, wherein two types of stripes are formed along one of the horizontal axis. These stripes consist of (TiO_2_)_5_ clusters, and they connect following a zig-zag pattern. Perpendicular to this arrangement, along another horizontal axis, we found thick linear links through oxygen atoms that give rise to hexagonal channels. These thick stripes join with others at an average distance of 3.65 Å (with a dispersion ranging from 3.59 to 3.77 Å). Within the flat platforms, triangular shapes emerge, formed by Ti–Ti bonds with an average value of 3.06 Å. Rectangular shapes with sides measuring 3.01 and 5.16 Å are also evident. The structural configuration shows an interplay of stripes, channels, and triangular motifs between Ti-Ti sites.

#### Using nearby isomer of (TiO_2_)_5_

Figure 5(ii) describes another bulk phase formed with a nearby isomer of the ground state. This structure consists of strips created by two (TiO_2_)_5_ clusters situated at different heigths, as shown in both panels (i) and (ii). These clusters are approximately the same size, and they exhibit an average Ti–Ti bond distance of 3.13 Å (with dispersion ranging from 3.03 to 3.31 Å). The Ti–Ti bonds between the clusters at different levels, while asymmetric, provide intriguing insights. A shorter Ti–Ti bond at 3.29 Å contrasts with a longer bond at 3.64 Å, both featuring a large width dispersion in the Ti-Ti distances. The structure features small hexagonal channels, adding to its complexity.

These structures are similar to previous cases resembling beehives, although with distinct arrangements for oxygen atoms. They exhibit various distortions especially in what concerns oxygen atoms. Furthermore, the energy gaps in these structures are nearly equal and comparable to those observed in the previous beehive cases, falling in the middle within the range of 2.15 to 2.70 eV.

#### Compact structure with a distinct isomer

 In Fig. 5(iii), we show the bulk structure resulting from the isomer illustrated in the (a) panel. This particular cluster yields a more regular structure characterized by significantly less dispersion in Ti-Ti distances, with an average value of 3.61 Å. The structure can be described as composed of bi-layers, presenting rectangular windows along one direction. In comparison to the previous two structures (Fig. 5(i,ii), which share similar densities ( 0.064 atoms/(Å)^3^). Although being less stable this configuration is more compact than the cases shown above. The energy gap, when computed with the same details, falls within the range of rutile and anatase phases. However, it is relevant to note that the structure appears considerably more densely packed than (i) and (ii) cases, offering a distinct perspective on the diverse range of structures attainable through self-assembly processes.

### Bulk phases with (TiO_2_)_6_ cluster as a seed

We last explore the bulk phases obtained by using different isomers of the (TiO_2_)_6_ cluster falling into the 0.1 eV from the most stable phase. Although these minima are local minima close to the ground state, none of they is the ground state, with the first two being planar and the last one adopting an octahedral configuration, as shown in panel (a) for each of the three cases.

#### Planar isomers and open cage structures

 In Fig. 6 (i) and (ii), we consider the formation of open cage structures that results from the first two planar isomers of (TiO_2_)_6_. The initial case starts with a configuration having the Ti-Ti skeleton in a square between two triangles. The resulting bulk is composed of parallel strips at varying heights, formed by (TiO_2_)_6_ clusters, each consisting of two triangles and a central square. These clusters are approximately the same size, with an average Ti-Ti distance of 3.11 Å. In the same line, the (TiO_2_)_6_ clusters are separated by 5.82 Å. The central squares exhibit an average Ti-Ti distance of 3.72 Å (dispersion ranging from 3.01 to 4.02 Å) within two flat-platforms at different levels. Additionally, asymmetric Ti triangles are observed with Ti-Ti distances of 3.13 Å (dispersion from 3.01 to 3.36 Å) and 3.56 Å (dispersion ranging from 3.18 to 3.75 Å), respectively.

In Fig. 6(ii), we found a bulk phase characterized by parallel stripes arranged in a zig-zag pattern along the vertical axis at various levels. These stripes are formed by (TiO_2_)_6_ clusters, each composed of four triangles in line. The clusters are nearly identical in size, with an average Ti-Ti distance of 3.08 Å. Within the same line, the clusters are situated 2.97 Å apart. Between different levels clusters are relaxed to be slightly assymetric, and the Ti-Ti separation averages to 3.86 Å for the upper part and 3.10 Å for the bottom part. This configuration shares similarities with the structures presented in Figs. 3(ii) and 4, although featuring larger plates derived from the initial cluster structure. These structures combine characteristics of bulk anatase with “T” layered structures, presenting a diverse interplay of features.

#### Octahedral-shaped seed

 Figure 6(iii) shows a bulk phase resulting from an input cluster seed with an octahedral shape. Unlike the previous two cases with two-dimensional Ti atom skeletons, this generating unit is remembered in the found three-dimensional bulk structure. The seed is an octahedron, consisting of Ti atoms decorated with O atoms bridging along the Ti-Ti edges. The relaxed bulk phase generated with these cluster seeds exhibits significantly higher density compared to the previous two configurations and shows square channels with octahedral Ti symmetry. The average Ti-Ti distance within the octahedra is 3.57 Å, with Ti-O distances measuring 1.956 Å. The structure obtained is highly regular, having large voids in the well-known TiO_2_ bulk structures. Anyhow this configuration is considerably much less stable, and it leads to stop our search for more open structures because the measured electronic properties of clusters seeds reach those of rutile and anatase bulk phases^47^.

## Discussion

In this discussion section, we summarize the findings from our computational study on TiO_2_ self-assembling clusters and their potential implications for the stability and electronic properties of TiO_2_-based structures. We aim to bridge the gap between the structural data, stability, and electronic properties to provide a comprehensive understanding of our results.

### Structural insights and stability-volume relationships

 As a result, we identified three distinct regions for the structures we examined:

i. Bulk-like Structures: This category includes well-established phases like rutile and anatase, well-known for their stability and prevalence in nature.
ii. Layered Structures: These structures, while intriguing, represent structures described in our previous investigation^64^. Previous research has already established the stability of layers similar to “T”-like structures and anatase one.
iii. Open Cage Structures: A remarkable finding is the emergence of a region characterized by hexagonal cage-like structures. These structures are reminiscent of open configurations such as zeolites and exhibit hexagonal and cubic-bulk patterns within the kinks.

**Figure 7:**
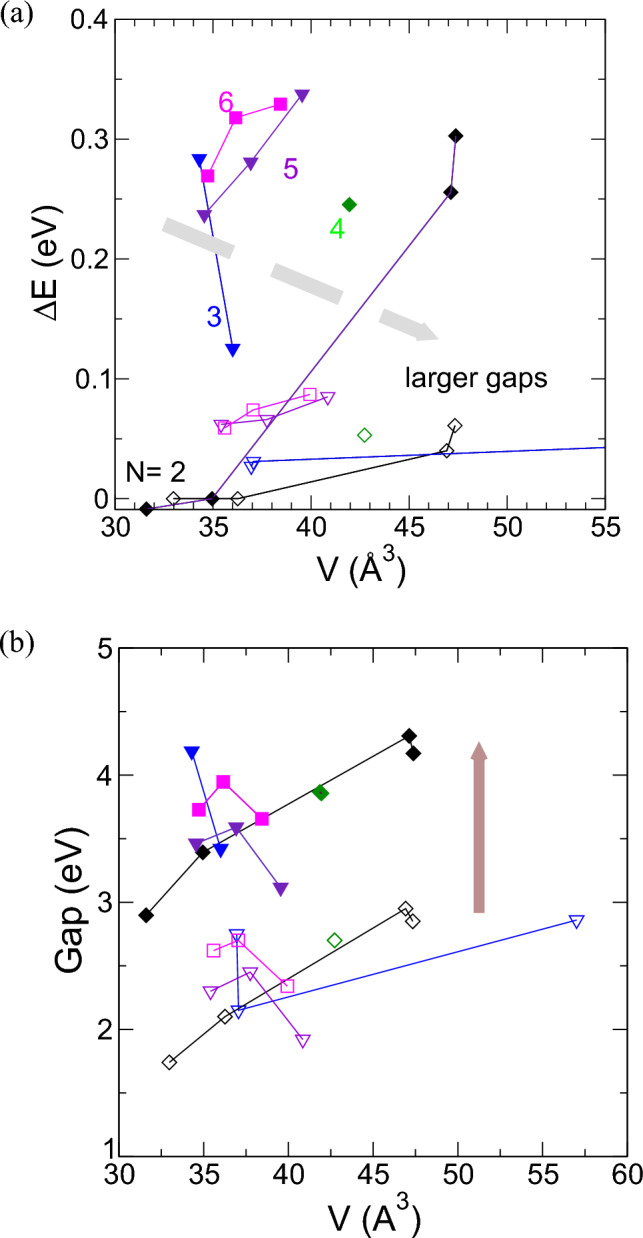
(**a**) Energy difference and (**b**) gaps versus volume of the phase structures generated using different clusters as seeds. The kind of seeds is indicated in the marks and joined with lines as indicated. Open symbols represent GGA results within 0.1 eV with respect to the stable anatase-rutile structures. Full marks denote corrected +U results that show larger energy differences and gaps that those obtained with GGA.

To synthesize the extensive data we have gathered, we then focused on plotting the energy differences per atom relative to the most stable structures against their volume. This approach allowed us to study stability and gaps versus an average of the structural data represented by volume, as shown in Fig. 7 with unfilled symbols. When increasing the volume we find that the energy differences with respect to the bulk rutile-anatase structures becomes larger, an expected trend that implies a decrease in stability.

**Figure 8:**
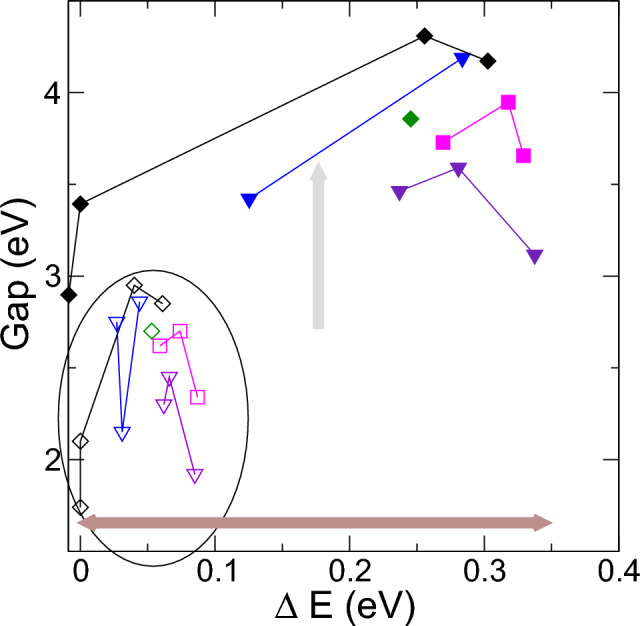
Gaps versus energy differences with respect to the stable anatase-rutile structures for the phase structures generated using different clusters as seeds. Filled and open symbols represent GGA and +U results, respectively. GGA results are enclosed with an ellipse: As shown above these results have smaller energy differences and gaps than +U results, although both data groups followed the same trends.

To further investigate the connection between stability and electronic properties, we also considered the energy gaps of the discussed structures against the energy differences, as shown in Fig. 8 with unfilled symbols. This allowed us to explore potential relationships between structural stability and electronic behavior. The gap trends align with the categorization of the structures into the aforementioned regions. We show that the gaps shown tend to be comparable or even higher than those of rutile and anatase for small stability energy differences till 0.2-0.3 eV. Gaps reach a limit and decrease following the large energy differences, i.e. when decreasing phase stability when N increases.

### Considerations for experimental validation

 Our computational study provides valuable insights into the stability and electronic properties of TiO_2_-based structures. However, it is needed to consider several factors when relating our results to experimental observations:

#### Structural validity

 Phonon calculations support the stability of rutile and anatase-like structures, both of which are found in nature. Additionally, layers resembling “T” structures and rutiles have been shown to be stable in prior research. As for zeolite-like structures, they are reasonable candidates for stability; however, further experimental validation is necessary. Experiments under low-density conditions should currently be pursued to explore these cases further.

#### Role of electron Coulomb interaction

 Our study underscores the significance of electron Coulomb interactions in transition metal oxides. Incorporating the Hubbard U parameter for both Ti and O plays a crucial role in accurately describing the energetic order of TiO_2_ phases^65,66^. This method enables the production of TiO_2_ phases in the right order of stability: rutile, anatase, and brookite. Including these U parameter leads to increased energy differences, implying enhanced stability, and is correlated with larger electronic band gaps. Then calculations with U were repeated for the GGA structures and relaxing inner and lattice parameters including volume, using VASP method^67–69^. These trends align with the amplified role of electron-electron interactions in transition metal oxides. Figures 7, 8 collect also the +U results computed from the GGA structures, now plotted with full symbols. We show that they followed similar trends but results using U parameters do not change volumes significantly and have lower stability with higher energy differences and gaps.

### Electronic properties of open structures

**Figure 9:**
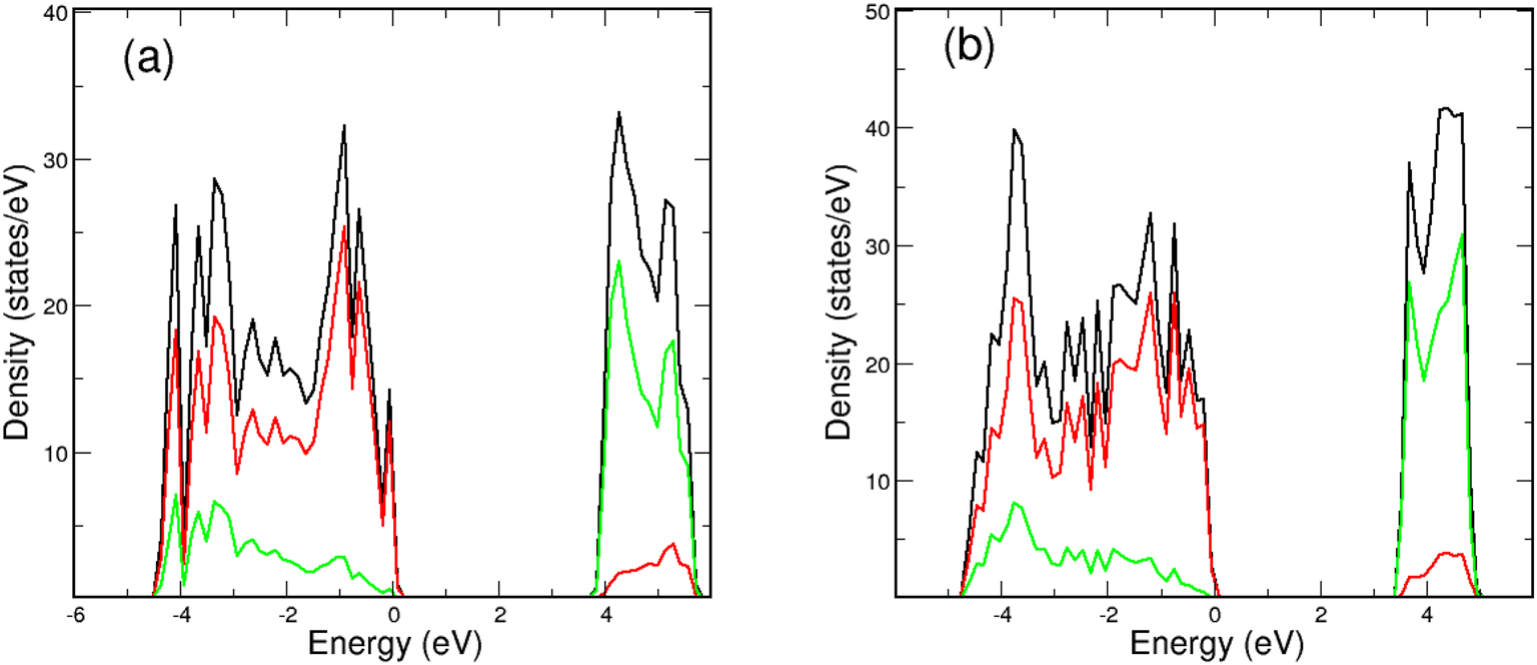
Density of states (DOS) versus energy for (**a**) zeolite-like and (**b**) beehive structures of the (i) panels in Figs. 4 and 5. The energy zero is set at the conduction band top. The DOS is plotted according to the constituent elements of oxygen and titanium plotted in red and green colors, respectively.

 In addition to elucidating the stability of the identified open structures, we present a comprehensive analysis of their electronic band structures, incorporating the Coulomb U parameter. The density of states (DOS) is shown in Fig. 9, using zeolite- and beehive-like structures as representative examples, displayed in the (i) panels of Figs. 4 and 5. Examining the (a) panel, it is evident that the DOS exhibits larger peaks around the valence band of zeolite-like structures. This observed patterns in the DOS are consistent across various other open structures. In contrast, the DOS for beehive structures shows a nearly flat plateau for both titanium (Ti) and oxygen (O) atoms, as depicted in the (b) panel, revealing remarkably similar patterns in all the cases. The DOS is separated based on the constituent elements, distinguishing between oxygen and titanium contributions. The local density of states (LDOS) for oxygen atoms is more pronounced in the valence band, where titanium atoms contribute predominantly in the conduction band. It is noteworthy that the titanium contribution is non-negligible in the lower region of the valence band, particularly for zeolite-like structures.

An intriguing observation is the narrowing of the conduction band in both open structures, resulting in the formation of a pseudogap shown above 5 eV within the conduction band. This distinctive feature underscores the unique electronic characteristics inherent in these open structures, providing valuable insights into their electronic properties. Furthermore, our findings reveal that these cage-like structures exhibit direct and substantial band gaps. The electronic properties of identified open structures in TiO_2_ can differ from traditional phases like anatase and rutile primarily because of having larger bandgaps. These unique properties are significant, suggesting potential applications in photocatalysis similar to those of anatase, both in bulk and nanoparticle forms. The larger band gaps observed in these cage structures indicate improved electronic properties compared to bulk anatase. In fact we also expect that atomic layer deposition on supported substrates may be a suitable approach for synthesizing these open phases, which could have been observed in preliminary studies^21,60,61^.

In summary, our comprehensive computational investigation provides valuable insights into the stability and electronic properties of TiO_2_-based structures. Note that the recent experiments included in Ref.^21^ provide empirical evidence on the stable thermodynamic conditions to synthesize the special TiO_2_ structures predicted by our density functional calculations. It paves the way for experimental validation of the newfound open structures, suggesting exciting prospects for advanced materials with applications in photocatalysis and beyond. Furthermore, the discovery of these open TiO_2_ phases resembling 2D transition metal chalcogenide layers holds promise for advancing materials science and technology, which opens new opportunities for device applications, catalysis, energy conversion, and fundamental science purposes.

## Methods and computational details

Density functional calculations identify stable TiO_2_ structures by minimizing total energy, optimizing atomic positions, and comparing formation energies to known rutile and anatase phases, facilitating materials discovery and design. For our computations, we follow a rigorous approach within Density Functional Theory (DFT) using the generalized gradient approximation (GGA), adopting the Perdew-Burke-Ernzerhof (PBE) formulation^70^. All calculations are performed within the framework of the SIESTA computational package, a versatile tool developed as part of the Spanish Initiative for Electronic Simulations with Thousands of Atoms^71^. SIESTA employs numerical pseudo-atomic orbitals as basis sets to solve the single-particle Kohn-Sham equations, with the atomic cores described by nonlocal norm-conserving Troullier-Martins pseudopotentials^72^, factorized in the Kleinman-Bylander form^73^. These pseudopotentials are generated, taking into account the valence configurations of 4s^2^3p^6^3d^2^ for Ti and 2s^2^2p^4^ for O. The cut-off radii for the s, p, and d orbitals are meticulously chosen, with Ti using a value of 1.98 atomic units (a.u.) for all orbitals, and oxygen s and p orbitals sharing a radius of 1.14 a.u. The valence states are described using double-doubly polarized basis sets. Detailed information regarding the pseudopotentials, basis sets used for impurities, and relevant tests can be found in our previous works^74–76^.

In our cluster calculations, we begin the process with three orthogonal lattice vectors, the sizes of which varied depending on the atomic base size used for each cell. These atomic base cells consist of two elementary (TiO_2_)_n_ clusters, acting as initial seeds, initially placed on a body center cubic (bcc) lattice array. This arrangement ensured that the interatomic distances among the atoms were at least comparable to those observed in the anatase and rutile bulk phases. In Fig. 3(iii), seeds are first relaxed facing each other to minimize the dipolar moment, and the resulting structure is used to form a closed packed bcc lattice with large distance in the z axis, and relaxed again. During the optimization process employing the conjugate gradient method^77^, the orthogonality of the lattice vectors may change without restriction on the lattice vectors and atomic positions of the atomic base. Sampling of the Brillouin zone involved a single $\Gamma$-point for geometry optimizations, and a 4 $\times$ 4 $\times$ 4 Monkhorst-Pack grid was employed for electronic structure calculations within the Brillouin zone integrations. A real-space grid for the electron density was defined with an energy cutoff of 250 Ry. Multiple tests were performed to validate the independence of the total energy values obtained for different energy cutoffs in the system.

With our calculations, we aim to investigate the effects of self-assembling on the finite size HOMO-LUMO gap, a critical aspect of our study that is further sheding light on the electronic properties of TiO_2_ cluster generated phases under specific conditions. The calculations include full spin polarization, and the structural and atomic relaxations are conducted without constraints during the optimization process till the forces are lower than 0.002 eV/Å, ensuring a comprehensive exploration of the impact of assembling TiO_2_ clusters.

### Supplementary Information

Supplementary Tables.

## Data Availability

All data generated or analysed during this study are included in this published article and its supplementary information files. Anyhow, the datasets used and/or analysed during the current study available from the corresponding author on reasonable request.
