# Supplementary material for: Low density phases of TiO2 by cluster self-assembly
Source: Sci Rep. 2024 May 31;14:12491. doi: 10.1038/s41598-024-61943-1 (PMC11143274; doi:10.1038/s41598-024-61943-1)
Supplement: Supplementary file 1 — Supplementary Tables. [file 41598_2024_61943_MOESM1_ESM.pdf]

* Corresponding author: a.ayuela@csic.es

Low Density Phases of TiO_2 by Cluster Self-Assembly

F. Aguilera-Granja[1,2] and Andres Ayuela[2,*]

[1] *Instituto de Física, Universidad Autónoma de San Luis Potosí, 78000 San Luis Potosí, México and*

[2] *Centro de Física de Materiales-CFM-MPC, Donostia International Physics Center DIPC, Paseo Manuel de Lardizabal 5, 20018 San Sebastin, Spain*

(Dated: March 25, 2024)

Abstract

For a comprehensive understanding of the structures reported in this work, we are including atomic coordinates, base vectors and their magnitude, volume, and atomic density. A detailed set of data is provided in tables included in this Supplementary Material. Reported structures are optimized within DFT at the GGA-level using the SIESTA method.

Keywords: DFT calculations, structural properties, electronic properties, self-assembly transition metal oxide clusters

TABLE 1. Some geometrial properties anatasa phase Fig. 2(i), atomic positions, lattice vectors, volume and atom density.

Element	X	Y	Z
Ti	-1.229296	-0.962672	0.010364
Ti	1.229708	0.964426	-0.009995
O	0.794717	-0.951573	-0.034826
O	-0.794307	0.954142	0.034489
O	-1.664893	-0.986961	-1.906542
O	1.665088	0.989430	1.906468
Ti	3.689197	0.946314	1.917341
Ti	6.148282	2.874686	1.897183
O	5.713298	0.958209	1.872215
O	4.124183	2.863691	1.941623
O	3.253793	0.922858	0.000588
O	6.583827	2.899485	3.813670
Vectors	X	Y	Z
**9.866**	9.866517	-0.013939	-0.020259
**3.834**	-0.015034	3.834081	0.000541
**3.834**	-0.014142	0.000381	3.834080
Vol. $(\AA)^3$	145.04 (0.0827)		

TABLE 2 . Some geometrial properties of the rutila phase Fig. 2(ii), atomic positions, lattice vectors, volume and atom density.

Element	X	Y	Z
Ti	-1.581940	0.001300	-0.272004
Ti	1.431477	0.005908	-0.277027
O	-0.044854	-1.220541	-0.353991
O	-0.034351	1.360454	-0.339032
O	2.968539	-1.215834	-0.359010
O	2.978921	1.365185	-0.344009
Ti	2.904475	-0.020869	3.007426
Ti	-0.108864	-0.025510	3.012546
O	1.466528	0.059413	1.667364
O	1.453161	0.073974	4.247768
O	-1.546786	0.054667	1.672386
O	-1.560129	0.069292	4.252737
Vectors	X	Y	Z
**6.027**	6.026832	0.009376	-0.010043
**4.668**	-0.000408	3.303098	3.298203
**4.688**	-0.011864	-3.317853	3.312958
Vol. $(\AA)^3$	131.90 (0.0913)		

TABLE 3. Some geometrial properties for the structure shown in Fig. 2(iii), atomic positions, lattice vectors, volume and atom density. Energy 0.04 eV/atom

Element	X	Y	Z
Ti	0.02790247	-0.34799271	0.51031453
Ti	-0.18829413	-3.36992435	-0.39693951
O	1.07070680	-0.11460369	1.96966851
O	0.97155673	-1.70017370	-0.56440558
O	-1.13825896	-2.01740858	0.66950489
O	0.41251190	1.06321362	-0.66423456
Ti	1.89650194	4.32296925	2.29811257
Ti	2.06915429	1.21889415	2.85228882
O	2.89085213	5.65888028	3.17961423
O	3.21482147	2.90518275	2.61633540
O	0.75659032	2.63449333	2.53358213
O	1.99384303	4.71294487	0.53561315
Vectors	X	Y	Z
**5.784**	4.945453	2.554788	1.574694
**9.839**	2.554871	9.498629	-0.244619
**5.513**	1.574447	-0.244852	5.278175
Vol. $(\AA)^3$		187.68 (0.0639)	

TABLE 4 . Some geometrial properties for the structure shown in Fig. 2(iv), atomic positions, lattice vectors, volume and atom density. Energy 0.061 eV/atom.

Element	X	Y	Z
Ti	-0.287995	0.177569	0.024914
Ti	-3.084525	-1.057458	-0.961013
O	1.441242	0.147299	-0.643587
O	-0.592266	2.006360	0.308158
O	-0.022151	-0.743703	1.615556
O	-1.126328	-0.709588	-1.466716
Ti	3.338607	2.299552	1.420577
Ti	6.132518	3.535991	2.406117
O	1.602531	2.326724	2.082863
O	3.635132	0.474608	1.133256
O	4.172635	3.186292	2.916290
O	3.067430	3.227764	-0.163183
Vectors	X	Y	Z
**6.143**	6.099250	0.319957	0.658418
**5.535**	0.342418	5.457411	-0.858640
**5.962**	0.584724	-0.747339	5.886505
Vol. $(\AA)^3$		188.95 (0.0635)	

TABLE 5. Some geometrial properties for the structure shown in Fig. 3(i), atomic positions, lattice vectors, volume and atom density. Energy 0.0275.

Element	X	Y	Z
Ti	0.30806693	-0.36023442	-2.63359128
Ti	1.83067214	1.52252962	-0.38577392
Ti	-1.21465356	1.52234427	-0.38102467
O	0.30918471	1.52669337	-1.99320495
O	3.35759156	1.51963993	0.66610138
O	-2.73370663	1.51941477	0.67518458
O	0.31196769	1.51953326	0.67159010
O	1.83550205	-0.36441602	-1.02588662
O	-1.21163761	-0.36460871	-1.02130316
Ti	4.86702234	1.50542491	6.05006898
Ti	6.39032130	-0.37715552	3.79757234
Ti	3.34410438	-0.37727898	3.80224887
O	4.87136640	-0.38145850	5.41038019
O	7.91178517	-0.37421438	2.74023574
O	1.82003169	-0.37437652	2.74943003
O	4.86584663	-0.37434111	2.74568501
O	6.39085052	1.50978692	4.43707618
O	3.34625655	1.50972341	4.44188919
Vectors	X	Y	Z
**9.177**	9.177120	0.000021	0.000761
**3.760**	0.000034	3.760441	0.048637
**6.424**	0.000826	0.048589	6.423912
Vol. $(\AA)^3$		221.67 (0.0812)	

TABLE 6. Some geometrial properties for the structure shown in Fig. 3(ii), atomic positions, lattice vectors, volume and atom density. Energy 0.0315 eV/atom.

Element	X	Y	Z
Ti	-0.83403123	0.02668208	-0.70891406
Ti	1.72467276	1.55026980	0.01636880
Ti	1.72350893	-1.50782601	0.01931302
O	1.09909636	0.02775193	-1.12447180
O	1.67988148	2.80220577	1.50686341
O	1.68003757	-2.75906646	1.50351201
O	2.35495154	0.02685636	1.16653582
O	-0.24165132	1.45489863	0.55098172
O	-0.23574089	-1.39625974	0.56041879
Ti	2.26380594	4.23707100	2.75084328
Ti	4.82233089	5.76019390	3.47561045
Ti	4.82117280	2.70210113	3.47854465
O	4.19696622	4.23784227	2.33484964
O	4.77765363	7.01283276	4.96589610
O	4.77791021	1.45115510	4.96315143
O	5.45271552	4.23712053	4.62599089
O	2.85606786	5.66510470	4.01011579
O	2.86189025	2.81388176	4.01981683
Vectors	X	Y	Z
**5.311**	5.109605	-0.002063	1.449027
**8.423**	0.001098	8.423853	-0.004269
**5.580**	1.084864	-0.001493	5.474125
Vol. $(\AA)^3$		222.38 (0.0809)	

TABLE 7. Some geometrial properties for the structure shown in Fig. 3(iii), atomic positions, lattice vectors, volume and atom density. Energy 0.102 eV/atom.

Element	X	Y	Z
Ti	-0.015727	-0.072690	-1.629495
Ti	1.940613	1.428720	0.296722
Ti	-2.023996	1.508430	0.263464
O	-0.007057	1.458709	-2.683425
O	3.825158	1.455560	0.815072
O	-3.898054	1.463497	0.775939
O	-0.034315	1.462972	-0.019426
O	1.902135	-0.057965	-1.001077
O	-1.957319	-0.050985	-1.027571
Ti	5.726443	1.420096	4.081661
Ti	7.733878	-0.107446	2.190288
Ti	3.767988	-0.101959	2.158869
O	5.720232	-0.092893	5.136063
O	9.608059	-0.066442	1.677177
O	1.884089	-0.065600	1.640498
O	5.743940	-0.065974	2.472535
O	7.669227	1.440963	3.479453
O	3.808174	1.440138	3.456441
Vectors	X	Y	Z
**11.570**	11.570108	-0.002473	0.060850
**3.034**	0.000413	3.034265	0.012526
**15.131**	0.127568	-0.058537	15.130420
Vol. $(\AA)^3$		531.17 (0.0339)	

TABLE 8 . Some geometrial properties for the structure shown in Fig. 4, atomic positions, lattice vectors, volume and atom density. Energy 0.053 eV/atom.

Element	X	Y	Z
Ti	-1.412409	-0.540615	-0.535306
Ti	0.408915	-2.238689	1.191575
Ti	-0.364087	2.247721	-1.394414
Ti	1.457241	0.559489	0.335336
O	0.731408	2.528642	0.106595
O	3.075525	0.896137	-0.541093
O	-3.030488	-0.866238	0.342817
O	2.096593	-1.234912	0.856487
O	-0.698207	-2.515879	-0.304082
O	-0.284266	-0.397062	1.055335
O	-2.055926	1.250876	-1.065799
O	0.331537	0.413513	-1.262358
Ti	1.042036	3.390306	1.867993
Ti	2.863361	1.692101	3.594638
Ti	2.090475	6.178497	1.008408
Ti	3.911832	4.490113	2.738022
O	3.185963	6.459241	2.509669
O	5.529916	4.826799	1.861994
O	-0.575673	3.064693	2.745652
O	4.551166	2.695592	3.259600
O	1.756328	1.414834	2.098884
O	2.170312	3.533662	3.458398
O	0.398562	5.181929	1.337239
O	2.785939	4.344142	1.140334
Vectors	X	Y	Z
**7.376**	7.108144	-1.318372	-1.467810
**8.983**	-1.318372	8.871659	0.513693
**5.835**	-0.880686	0.308216	5.760136
Vol. $(\mathring{A})^3$		341.83 (0.0702)	

TABLE 9. Some geometrial properties for the structure shown in Fig. 5(i), atomic positions, lattice vectors, volume and atom density. Energy 0.0625 eV/atom.

Element	X	Y	Z
Ti	-1.50671300	-0.54967681	-0.12960984
Ti	1.50757216	-0.54894057	-0.13482793
Ti	0.00048081	2.04637601	0.26321410
Ti	-3.10693417	2.06906006	0.06703912
Ti	3.10724942	2.07013740	0.05947014
O	-1.51357793	2.88263711	-0.91550669
O	1.51206087	2.88307106	-0.91969246
O	-4.31625155	1.16498746	1.28708994
O	4.31999872	1.16747334	1.27863601
O	-2.96490661	0.32365406	-1.09364523
O	2.96448546	0.32505174	-1.10127845
O	0.00280596	-1.36580250	1.00579786
O	-0.00077119	0.34257242	-0.97660312
O	-1.54779630	1.14988191	0.99258055
O	1.55140470	1.14996785	0.98795833
Ti	5.76092551	2.03437260	2.49422152
Ti	8.77578858	2.03551186	2.49414546
Ti	7.26747150	4.62752162	2.09292063
Ti	4.16048408	4.64746423	2.32767858
Ti	10.37459160	4.64915072	2.32783266
O	5.75645797	5.46259282	3.28266664
O	8.77980351	5.46336267	3.28238861
O	2.94929057	3.75014181	1.08661361
O	11.58592867	3.75260387	1.08469783
O	4.30582864	2.90709921	3.46679357
O	10.23177097	2.90841634	3.46651093
O	7.26888568	1.21761300	1.35484295
O	7.26836976	2.92450530	3.33917629
O	5.71695186	3.73009402	1.37533237
O	8.81883428	3.73068351	1.37520717
Vectors	X	Y	Z
**14.532973**	14.532969	0.003399	-0.009620
**5.163298**	-0.001112	5.163298	0.001688
**4.718592**	0.003307	0.001352	4.718591
Vol. $(\AA)^3$		354.07 (0.0847)	

TABLE 10. Some geometrial properties for the structure shown in Fig. 5(ii), atomic positions, lattice vectors, volume and atom density. Energy 0.066 eV/atom.

Element	X	Y	Z
Ti	-2.758178	-0.553900	0.445257
Ti	0.305250	-0.730004	0.370375
Ti	-1.172085	2.049327	-0.026717
Ti	1.764611	1.860993	-0.831704
Ti	3.393280	-0.845443	-0.307375
O	-1.977795	3.435536	1.020326
O	0.493293	2.946404	0.246312
O	3.431791	0.902412	-1.476917
O	1.996007	0.356439	0.667706
O	-1.246888	-1.692447	-0.376308
O	1.667842	-1.703377	-0.740393
O	-1.098009	0.454657	1.094798
O	-3.091919	1.026675	-0.407579
O	0.202248	0.868196	-1.107693
O	4.130251	-2.315627	0.571956
Ti	3.115091	1.268727	2.164117
Ti	6.057847	1.143662	1.400357
Ti	4.593978	3.879934	0.960337
Ti	1.513244	3.988431	1.636146
Ti	7.655994	3.752968	0.917504
O	3.238887	4.843660	2.073193
O	6.151882	4.873547	1.719302
O	0.763761	5.467082	0.763271
O	1.466504	2.236323	2.807853
O	7.967556	2.170674	1.788451
O	4.410519	0.204795	1.091539
O	6.897422	-0.240413	0.347949
O	4.675946	2.287570	2.438949
O	2.904978	2.790494	0.652443
O	6.003698	2.717951	0.249893
Vectors	X	Y	Z
**10.658**	10.524234	0.172561	-1.678613
**7.255**	0.122591	7.167933	-1.117885
**5.312**	-0.716653	-0.671987	5.221184
Vol. $(\AA)^3$		377.50 (0.0795)	

TABLE 11. Some geometrial properties for the structure shown in Fig.5(iii), atomic positions, lattice vectors, volume and atom density. Energy 0.085 eV/atom.

Element	X	Y	Z
Ti	-1.565415	-0.565553	0.303201
Ti	1.650209	-0.565740	0.303795
Ti	0.042175	2.038469	-0.543355
Ti	-2.984532	2.280756	-1.031889
Ti	3.068941	2.280543	-1.031359
O	-1.391258	3.279940	-0.245898
O	1.475775	3.279749	-0.244945
O	-3.155030	3.441204	-2.469310
O	3.238417	3.441037	-2.468943
O	-2.829875	1.060664	0.622522
O	2.915146	1.060151	0.622872
O	0.042318	-1.465189	-0.010897
O	0.042645	0.874413	0.811497
O	-1.357265	1.053830	-1.589048
O	1.442164	1.054061	-1.588387
Ti	3.849666	1.826216	2.082482
Ti	7.065414	1.826157	2.082886
Ti	5.457374	4.430439	1.235585
Ti	2.430585	4.672586	0.747220
Ti	8.484194	4.672498	0.747678
O	4.023860	5.671845	1.533248
O	6.890965	5.671655	1.534212
O	2.260091	5.832997	-0.690206
O	8.653533	5.832927	-0.689665
O	2.585004	3.452456	2.401526
O	8.330103	3.452127	2.402149
O	5.457519	0.926895	1.767955
O	5.457311	3.266554	2.590521
O	4.057792	3.445841	0.189775
O	6.857374	3.445849	0.190727
Vectors	X	Y	Z
**10.830**	10.829976	-0.000483	0.000869
**7.681**	-0.000386	7.265494	-2.481260
**6.528**	0.000695	-2.481260	6.038622
Vol. $(\AA)^3$	408.47 (0.0735)		

TABLE 12 . Some geometrial properties for the structure shown in Fig.6(i), atomic positions, lattice vectors, volume and atom density. Energy 0.059 eV/atom.

Element	X	Y	Z
Ti	-2.184430	-1.016831	0.299173
Ti	0.650553	-1.535258	-0.704822
Ti	-0.642889	1.551223	0.745534
Ti	-3.885270	1.523460	0.818667
Ti	2.189813	1.036390	-0.203722
Ti	3.898288	-1.505927	-0.773031
O	-2.021100	2.197452	1.877908
O	1.206671	1.827228	1.247921
O	-5.220468	1.099722	-0.330750
O	3.545881	0.243117	-1.502037
O	-3.542137	-0.221322	1.580082
O	2.301056	-0.861520	0.501233
O	-1.204656	-1.809877	-1.169353
O	2.053699	-2.169929	-1.813540
O	-0.644585	-0.379884	1.104863
O	-2.290901	0.877975	-0.434073
O	0.643375	0.398609	-1.014573
O	5.237961	-1.083985	0.376128
Ti	2.878983	2.468022	2.468990
Ti	5.715334	1.948313	1.463703
Ti	4.420561	5.035894	2.916446
Ti	1.178491	5.008956	2.988416
Ti	7.253807	4.520720	1.964922
Ti	8.962684	1.978399	1.395314
O	3.043402	5.684234	4.047715
O	6.270848	5.311085	3.416804
O	-0.156244	4.584467	1.838427
O	8.609154	3.726218	0.665512
O	1.522207	3.264679	3.750456
O	7.365087	2.622347	2.669617
O	3.859266	1.675032	1.000651
O	7.118117	1.309815	0.357185
O	4.418976	3.105796	3.274896
O	2.773103	4.363379	1.736166
O	5.709009	3.881644	1.152827
O	10.301739	2.401478	2.544967
Vectors	X	Y	Z
**12.003**	11.786071	-1.141599	-1.964357
**7.555**	-0.815428	7.423686	1.145659
**5.270**	-0.841867	0.687395	5.157299
Vol. $(\AA)^3$	427.09 (0.0843)		

TABLE 13. Some geometrial properties for the structure shown in Fig. 6(ii), atomic positions, lattice vectors, volume and atom density. Energy 0.074 eV/atom.

Element	X	Y	Z
Ti	-2.150488	-1.648289	0.070441
Ti	0.849220	-0.966581	-0.525898
Ti	-1.191029	1.188776	0.569117
Ti	-4.246314	0.382570	0.633077
Ti	1.789234	1.888978	-0.073566
Ti	3.899329	-0.159119	-0.594022
O	-2.710164	1.438264	1.697372
O	0.145827	2.371173	1.226551
O	-5.515848	-0.351435	-0.581217
O	3.175715	1.430316	-1.205300
O	-3.518421	-1.221234	1.228044
O	2.256730	-0.048919	0.722390
O	-0.483469	-2.147297	-1.219758
O	2.366390	-1.240547	-1.652592
O	-0.716381	-0.613163	0.930661
O	-2.598204	0.260954	-0.690262
O	0.367089	0.834314	-0.908676
O	5.174822	0.575768	0.607792
Ti	6.322291	1.877335	1.681602
Ti	9.321253	2.558839	1.085626
Ti	7.281852	4.714246	2.179802
Ti	4.225952	3.908112	2.243817
Ti	10.261533	5.414513	1.537348
Ti	12.370774	3.366175	1.017215
O	5.762483	4.963975	3.307533
O	8.618542	5.897019	2.837042
O	2.956462	3.173964	1.030628
O	11.648222	4.955725	0.405339
O	4.954053	2.304084	2.838402
O	10.729645	3.476713	2.332314
O	7.988520	1.378635	0.391982
O	10.839385	2.285758	-0.041176
O	7.756298	2.912534	2.540978
O	5.873856	3.786744	0.920696
O	8.838800	4.360193	0.702812
O	13.647613	4.101167	2.218044
Vectors	X	Y	Z
**16.283**	16.090277	1.238155	-2.174415
**5.970**	1.719185	5.709464	0.310459
**5.159**	-0.864643	0.103498	5.085742
Vol. $(\mathring{A})^3$	444.42 (0.0810)		

TABLE 14. Some geometrial properties for the structure shown in Fig. 6(iii), atomic positions, lattice vectors, volume and atom density. Energy 0.087 eV/atom.

Element	X	Y	Z
Ti	1.71881154	1.44097352	-0.03313668
Ti	1.67013010	-2.00222168	-0.63110781
Ti	-1.65888734	1.89902217	0.63970968
Ti	-1.70649724	-1.54456874	0.04230541
Ti	0.42107527	-0.90320276	2.49369731
Ti	-0.40704150	0.79955040	-2.48164006
O	1.24192261	0.63923283	1.66115430
O	1.64033496	-2.01267721	3.13434492
O	-1.00763626	2.40932206	2.26178428
O	-1.15458116	-1.35747963	3.44584421
O	1.16774945	1.25194904	-3.43673896
O	1.02221419	-2.51248285	-2.25563977
O	-1.62022253	1.91442689	-3.12467902
O	-1.23110588	-0.74333374	-1.65294088
O	1.99678592	-0.17344026	-0.88511525
O	-1.98482282	0.06940994	0.89328335
O	-0.12070420	1.57650731	-0.66774728
O	0.13404912	-1.68009072	0.67569660
Ti	4.48583277	5.21879283	2.92234613
Ti	4.43784876	1.77602832	2.32441169
Ti	1.10890584	5.67642610	3.59574609
Ti	1.06105192	2.23291871	2.99811338
Ti	3.18866649	2.87442491	5.44898060
Ti	2.35960765	4.57711972	0.47323560
O	4.00974923	4.41676401	4.61689812
O	4.40794135	1.76507470	6.09063887
O	1.75931189	6.18609965	5.21869955
O	1.61331243	2.41996583	6.40166767
O	3.93440056	5.03020177	-0.48171617
O	3.78979305	1.26470263	0.70011854
O	1.14252982	5.68835533	-0.16948884
O	1.53870882	3.03376576	1.30337447
O	4.76431934	3.60433362	2.07031291
O	0.78359230	3.84688118	3.84924175
O	2.64636038	5.35414675	2.28778726
O	2.90184759	2.09618260	3.63162109
Vectors	X	Y	Z
**7.384**	7.263180	-1.037249	-0.837758
**9.264**	-0.952433	9.189706	-0.691928
**7.505**	-0.775429	-0.597448	7.441158
Vol. $(\AA)^3$		479.31 (0.0751)	
